# The Efficacy of Transarterial Embolization for Postpartum Hemorrhage Complicated with Disseminated Intravascular Coagulation: A Single-Center Experience

**DOI:** 10.3390/jcm10184082

**Published:** 2021-09-09

**Authors:** Daigo Ochiai, Seishi Nakatsuka, Yushi Abe, Satoru Ikenoue, Yoshifumi Kasuga, Masanori Inoue, Masahiro Jinzaki, Mamoru Tanaka

**Affiliations:** 1 Department of Obstetrics and Gynecology, Keio University School of Medicine, Tokyo 160-8582, Japan; y.abe@keio.jp (Y.A.); sikenoue.a3@keio.jp (S.I.); 17yoshi23.k@gmail.com (Y.K.); mtanaka@keio.jp (M.T.); 2 Department of Diagnostic Radiology, Keio University School of Medicine, Tokyo 160-8582, Japan; nakatsuka@rad.med.keio.ac.jp (S.N.); inomasa@mac.com (M.I.); jinzaki@rad.med.keio.ac.jp (M.J.)

**Keywords:** transarterial embolization, postpartum hemorrhage, disseminated intravascular coagulation, uterine atony, placenta accreta spectrum, placenta previa

## Abstract

Indications for the use of transarterial embolization (TAE) for postpartum hemorrhage (PPH) have been established. However, the efficacy of TAE for PPH complicated by disseminated intravascular coagulation (DIC) remains controversial. In this study, we investigated the efficacy of TAE for PPH complicated by DIC. A database review was conducted to identify patients who were treated with TAE for PPH at our hospital. TAE was performed in 41 patients during the study period. Effective hemostasis was achieved in all cases, but additional procedures, such as re-embolization or hysterectomy, were required in five patients (12.2%). The typical causes of PPH included uterine atony (18 cases), placenta previa (15 cases), amniotic fluid embolism (DIC-type) (11 cases), and placenta accreta spectrum (10 cases). The mean blood loss was 3836 mL. The mean obstetrical DIC and the International Society on Thrombosis and Hemostasis DIC scores were 7.9 and 2.6, respectively. The efficacy of hemostasis was comparable between patients with and without DIC. However, the complete success rate of TAE was lower in patients with DIC as the condition worsened than that in non-DIC patients. Overall, TAE is effective as a minimally invasive treatment for PPH complicated by DIC.

## 1. Introduction

Postpartum hemorrhage (PPH), a pregnancy complication that may occur during or after delivery, is one of the most common causes of maternal morbidity and mortality [1,2]. PPH is initially treated with rehydration, blood transfusion, and oxytocin administration, followed by local procedures such as uterine balloon tamponade within the uterine cavity. However, if bleeding continues, surgical procedures (e.g., hysterectomy and internal iliac artery ligation) or transarterial embolization (TAE) may be required for management.

TAE, including uterine artery embolization, has been attracting attention in the field of interventional radiology as a treatment option for PPH. TAE reportedly results in shorter hospital stays, requires a smaller blood transfusion volume, and may preserve fertility compared with hysterectomy [3,4,5]. Although there are valid concerns regarding the effects of TAE on women who wish to retain fertility, pregnancy after this procedure is well documented [6,7,8]. In general, pregnancy after TAE is possible without significant morbidity or mortality. According to the Royal College of Obstetricians and Gynecologists and the Japanese Society of Interventional Radiology guidelines, TAE should be considered in the treatment of PPH [9,10]. The indications for the use of TAE for PPH have been established [11,12,13,14,15]. However, the utility of TAE for PPH complicated by disseminated intravascular coagulation (DIC) remains controversial [13,16,17,18,19].

In this study, we investigated the efficacy of TAE for PPH complicated with or without DIC in our hospital.

## 2. Materials and Methods

A database review was conducted to identify patients who were treated with TAE for PPH after delivery at Keio University Hospital between 1 September 2012 and 31 June 2021. Opt-out consent was obtained from patients due to the retrospective design of this study. The study was approved by the Research Ethics Review Board of Keio University (No. 20150103).

Antenatal data, such as maternal demographic information (age, body mass index parity, mode of conception, number of gestations, and obstetric complications), were collected retrospectively. Diagnoses of hypertensive disorders of pregnancy, gestational diabetes mellitus, and placenta previa were based on the clinical criteria of the Japan Society of Obstetrics and Gynecology [20]. Delivery information, including gestational age at delivery, mode of delivery, and additional procedures after TAE to control bleeding, were reviewed. Moreover, we collected details about the PPH, including the etiology of hemorrhage and the amount of blood transfusion provided. Total blood loss was defined as the total volume of blood loss from delivery to TAE. To evaluate the contribution of TAE to improvements in hemodynamics, complete success rate and hemostasis efficacy after TAE were investigated. Complete success was defined as hemostasis achieved by initialTAE alone, requiring no additional hemostatic interventions. In contrast, the efficacy of hemostasis was determined according to the number of cases in which patients showed a decrease in bleeding, a stabilization of patient’s condition, or a decrease in DIC scores, but underwent re-embolization or hysterectomy resulting in complete success [17]. The diagnostic criteria for DIC were based on the obstetrical DIC score [21] and the International Society on Thrombosis and Hemostasis (ISTH) score [22]. Patients were diagnosed with DIC according to three diagnostic criteria: obstetrical DIC score ≥ 8, obstetrical DIC score ≥ 13, or ISTH score ≥ 5. Amniotic fluid embolism (AFE) was defined based on the Japan consensus criteria for the diagnosis of AFE, which was based on the United States/United Kingdom criteria and was divided into DIC-type and cardiopulmonary collapse type [23]. PPH occurring within 24 h of delivery was classified as primary PPH, while PPH occurring at least 24 h after delivery, but within 6 weeks postpartum, was classified as secondary PPH [24]. The placenta accreta spectrum (PAS) refers to the range of pathologic adherence of the placenta, including placenta increta, placenta percreta, and placenta accreta [25].

All TAE procedures were performed by an interventional radiologist using an angiography system under local anesthesia with lidocaine. Based on the information obtained from the obstetrician before TAE, specifically regarding the pathology causing the hemorrhage and the observations on angiography, a microcatheter was inserted into the artery supplying the bleeding site, and feeding vessels were selectively embolized according to the observations of angiography using gelatin sponges (Serescue, Astellas Pharma Inc., Tokyo, Japan), NBCA (N-butyl-2-cyanoacrylate) (Histoacryl, Aesculap AG, Tuttingen, Germany), and metallic coils.

IBM SPSS 25 statistical software (SPSS Inc., Chicago, IL, USA) was used for the statistical analyses. The Mann–Whitney U test was used to analyze continuous variables, and the Fisher’s exact test was used to analyze intergroup differences for categorical data. The level of statistical significance was set at *p* < 0.05.

## 3. Results

### 3.1. Patient Characteristics

TAE was performed 43 times for the 41 patients with PPH, including 2 patients who underwent the procedure twice during the study period. Table 1 shows the patient characteristics. The mean maternal age was 37.3 years (range: 29–45 years). Among the 41 patients, 33 patients (78.6%) were primiparous. The mean gestational age at delivery was 35.4 weeks (range: 30.0–41.4 weeks). In terms of the mode of delivery, there were 4 cases of spontaneous vaginal delivery, 4 cases of instrumental delivery, and 33 cases of cesarean section (CS). The mean blood loss was 3836 mL (range: 1300–10,000 mL), and the typical causes of PPH included uterine atony (18 cases), placenta previa (15 cases), amniotic fluid embolism (DIC-type) (11 cases), PAS (10 cases), retained products of conception (RPOC) (4 cases), pseudoaneurysm (4 cases), vaginal hematoma (1 case), and placental abruption (1 case). The mean obstetrical DIC score was 7.9 (range 0–24), and the number of cases with a score of ≥8 or ≥13 was 19 (46.3%) and 9 (22.0%), respectively. The mean ISTH-DIC score was 2.6 (range: 0–8), and the number of cases with a score of ≥5 was 11 cases (26.8%). Blood transfusion was performed in all patients. The mean units of red blood cells (RBC), fresh frozen plasma (FFP), and platelet concentrates (PC) transfused in patients were 12.0 (range: 0–40), 12.6 (range: 0–59), and 11.5 (range: 0–90), respectively.

A summary of the TAE findings is shown in Table 2. Bilateral and unilateral uterine artery embolization was performed in 38 and 5 patients, respectively. Vessels other than the uterine artery were embolized in 12 cases, and the embolized vessels were the internal pudendal, internal obturator, internal bladder, inferior gluteal, superior vesical, inferior vesical, inferior epigastric, superior gluteal, and ovarian arteries. Gelatin sponge was the most favored embolic agent (34 cases). NBCA and coil were used in seven and two cases, respectively. Hemostasis was achieved in all the cases. Among them, complete success was not achieved in five patients in which re-embolization (two cases) or hysterectomy (three cases) were required for further management.

### 3.2. The Characteristics of Patients with or without Obstetrical and ISTH DIC

Table 3 shows the characteristics of patients with or without obstetrical and ISTH DIC. We defined DIC as an obstetrical DIC score ≥ 8, obstetrical DIC score ≥ 13, and ISTHDIC score ≥ 5. We performed the following analyses according to the presence or absence of DIC in the three different definitions. There were no significant differences in patient characteristics such as maternal age, body mass index, parity, mode of conception, number of fetuses, and number of prior CS between groups with or without DIC using any of the three definitions. 

### 3.3. The Obstetric Outcomes in Patients with or without Obstetrical and ISTH DIC

Table 4 shows the obstetric outcomes of patients with or without DIC. There were no significant differences in gestational age at delivery or mode of delivery between the two groups. However, the mean total blood loss and the requirement for blood transfusion (RBC, FFP, and PC) were significantly higher in patients with either obstetrical DIC or ISTH DIC than in those without DIC. The FFP/RBC ratio was comparable between the two groups, and the ratio was approximately 1 or higher. Primary PPH was more common in the DIC group than in the non-DIC group, and these differences were statistically significant when DIC was defined as an obstetrical DIC score ≥ 8.

### 3.4. PPH Etiology, Embolic Materials used, and Clinical Outcomes Patients who underwent TAE with or without Obstetrical and ISTH DIC

Table 5 shows the PPH etiology and embolic materials used for TAE in patients with or without DIC. The etiology of PPH demonstrated differing characteristic features between the DIC and non-DIC groups. In the DIC group, uterine atony and AFE (DIC-type) were more frequently observed. In contrast, placenta previa and PAS were lower in the DIC group than in the non-DIC group. The embolic material varied according to the presence or absence of DIC. Gelatin was more frequently used in all patients, while NBCA were significantly selected more in the DIC group.

Table 6 shows the clinical outcomes of patients who underwent TAE with or without DIC. The efficacy of hemostasis was comparable between patients with or without DIC diagnosed using any of the diagnostic criteria. Moreover, in the early stages of DIC, the complete success rate of TAE was comparable between patients with and without DIC, but was lower as obstetrical DIC worsened in DIC patients than that in non-obstetrical DIC patients.

## 4. Discussion

The effectiveness of TAE in PPH has been reported in many studies [13,15,19,26,27,28]. However, there are conflicting reports on the efficacy of TAE for PPH complicated by DIC [19,21,22,29]. In this institutional study, we found that TAE was a useful strategy for managing PPH regardless of the presence or absence of DIC. Hemostatic improvement was achieved in all patients, and the overall complete success rate was 87.8% upon treatment with TAE. We also observed that the complete success rate of TAE was lower as obstetrical DIC worsened compared to that in non-obstetrical DIC patients. Additionally, we also found a higher volume of blood transfusion for hemodynamic improvement and coagulation factor replacement in the DIC group compared to the non-DIC group. Further, NBCA use could contribute to reliable hemostasis in patients with DIC.

To date, TAE is one of the most effective treatments for PPH and is superior to hysterectomy because it is less invasive and preserves fertility. However, its efficacy remains unclear in patients with unstable hemodynamics and coagulopathies, which are typical features of DIC [19,21,22,29]. The clinical success rate of TAE in hemostasis was reported to be approximately 90% [13,15,26,27,28], which is comparable to our results. In this study, we investigated the utility of TAE in patients with or without DIC according to the three diagnostic criteria for DIC. As a result, we found that TAE is a useful hemostatic technique for obstetric PPH with or without DIC, irrespective of the DIC criteria used (Table 4 and Table 5). The diagnostic criteria for DIC included the obstetrical and ISTH DIC criteria [21,22]. The former is frequently used in Japan, with a score that focuses on the underlying disease and clinical symptoms. Using the obstetrical DIC score, patients are diagnosed with DIC above 13 points, but it is recommended to start DIC treatment when the score is above 8 points [21]. In contrast, the latter diagnostic criterion for DIC is based only on the numerical values of blood tests. This ISTH diagnostic criterion is objective and is frequently used in the field of trauma, where DIC is diagnosed at >5 points [22]. The efficacy of hemostasis was comparable between patients with or without DIC diagnosed using any of the three criteria. As a result of using three different diagnostic criteria for DIC, we found that the complete success rate of TAE was comparable between patients with or without DIC in the early stages, but was lower as obstetrical DIC worsened in DIC patients than that in non-obstetrical DIC patients.

Obstetric PPH is characterized by the development of intractable DIC in a short period of time [21,29]. It has been reported that unstable hemodynamics and coagulopathies in DIC could lead to a failure of TAE for PPH and an increase in complications resulting from TAE [12,18,26,27,30,31,32]. In previous reports of large-scale surveys, the success rate of TAE for PPH in patients with DIC has been reported to be approximately 62.5–75.8%, which is lower than that in patients without DIC [13,27]. Although the present study was a single-center, retrospective study, the efficacy of hemostasis and complete success rate of TAE in patients with DIC was approximately 100% and 66.7–84.2%, respectively, which was better than previously reported [12,18,26,27,30,31,32]. Compared with previous reports, this study involved massive blood transfusions for patients in the DIC group. Since rapid replacement of coagulation factors can provide a better hemostatic effect, especially in DIC, massive blood transfusion might contribute to the higher therapeutic effect of TAE in DIC. Furthermore, transfusion therapy with an FFP/RBC ratio of ≥1.0 has been recommended for the treatment of obstetrical DIC [29,33]. In our study, the FFP/RBC ratio was approximately 1.0 or higher, which was comparable between the DIC and non-DIC groups (Table 4). These results suggest that, when TAE is performed for DIC-complicated PPH, it is crucial to perform massive transfusion while maintaining an appropriate FFP/RBC ratio together with TAE to improve unstable hemodynamics and coagulopathies in DIC.

According to previous reports, risk factors for unsuccessful TAE include primiparity, extravasation, and anatomical variations in the uterine arteries [12,18,19,26,27,30,31]. In our study, five patients (12.2%) required additional procedures (re-embolization, two cases; hysterectomy, three cases). In cases that required re-embolization, complete hemostasis was considered in the initial TAE using NBCA. Although there was slight extravasation, the hemodynamics improved significantly during the procedure. To avoid post-embolization complications, such as uterine necrosis and menstrual abnormalities, the patient was observed without NBCA until the next day, and TAE using gelatin sponge with or without metallic coil was performed upon the progression of anemia (Cases 5 and 7). The selection of embolization material is an important factor in the treatment of PPH complicated by DIC. One of the mechanisms by which gelatin sponges blocks blood flow is through clot formation around the substance, thus requiring the host’s coagulation capacity for successful embolization. In contrast, NBCA is a permanent embolic material that has the potential for immediate hemostasis independent of coagulation factors. However, NBCA may cause post-embolization complications, such as uterine necrosis and menstrual abnormalities, more frequently than gelatin sponges, but gelatin sponge cut into small pieces has been reported to cause the same complications [34,35]. When performing TAE for PPH complicated by DIC, gelatin sponge that is not too fragmented should be the first choice for the embolic material. When hemostasis is difficult, NBCA together with a gelatin sponge may be more optimal. When the initial TAE with gelatin sponge can stabilize the circulation, it may be possible to perform an additional TAE using only a gelatin sponge to reduce the possibility of post-embolization complications.

In the present study, there were three cases in which hysterectomy had to be performed following TAE. In Case 8, a cesarean section was performed because of pregnancy after trachelectomy for early-stage cervical cancer, and secondary PPH due to postoperative RPOC was completely stopped by TAE. However, the patient (Case 8) was forced to undergo hysterectomy due to intractable abdominal pain, probably due to post-embolization complications. In Case 18, the placenta was partially removed during the cesarean section, leading to massive bleeding. TAE was performed immediately in the operating room. However, the bleeding from the separated placenta was still intense and continued, resulting in a hysterectomy. In Case 24, an intrauterine balloon was immediately inserted for temporary hemostasis after cesarean section due to the onset of HELLP syndrome, and TAE was performed as the following procedure. When the balloon was removed after TAE, venous uterine bleeding and hemodynamic instability was observed, prompting emergency hysterectomy to control the bleeding. Previous reports have reported that the incidence of cases requiring hysterectomy after TAE for PPH is 4–12% [10,13,36], which is consistent with our study (7.3%, 3/41 patients). Thus, it is pertinent to keep in mind that TAE for PPH may require additional procedures, such as re-embolization or hysterectomy, and may cause post-embolization complications, such as fever and abdominal pain.

In our hospital, TAE for PPH exhibited hemostatic effect of 100% and an overall complete success rate of 87.8% (36/41 patients). Although there are some limitations in this study, such as the small number of cases at a single institution and the retrospective study design, our study indicated that TAE could be an effective life-saving strategy for PPH—a major cause of maternal death. In this study, the presence or absence of DIC did not affect the hemostatic efficacy of TAE, but the complete success rate of TAE was lower in DIC patients as the condition worsened than that in non-DIC patients. These results suggest that TAE is an effective minimally invasive treatment for PPH complicated by DIC when combined with adequate coagulation factor replacement.

## Figures and Tables

**Table 1:** Patient characteristics.

Case	Age	Parity	Etiology of PPH	Mode of Delivery	GA at Delivery	Blood Loss (mL)	Obstetrical DIC	ISTH DIC	RBC Units	FFP Units	PC Units
1	38	0	Placenta accreta spectrum	CS	37–2	1610	5	0	0	0	0
2	37	0	Vaginal hematoma	I-VD	39–1	2540	6	0	8	8	0
3	37	0	Pseudoaneurysm	I-VD	40–2	2100	0	0	4	0	0
4	37	0	Uterine atony	CS	39–0	3000	6	6	12	7	20
5	38	3	Placenta accreta spectrum, Placenta previa	CS	33–2	5500	15	3	30	25	20
6	38	2	Placenta previa	CS	36–5	4240	8	2	6	8	0
7	39	0	Amniotic fluid embolism (DIC-type), Uterine atony	CS	36–1	5700	15	3	16	6	20
8	38	0	Retained products of conception	CS	37–1	2600	0	0	4	0	0
9	29	0	Placenta accreta spectrum	S-VD	39–1	1850	5	0	10	0	0
10	38	1	Placenta previa, Uterine atony	CS	30–0	3000	5	2	8	10	0
11	36	0	Uterine atony	S-VD	41–3	4000	8	0	12	5	0
12	32	0	Amniotic fluid embolism (DIC-type), Uterine atony	CS	37–6	4100	13	5	20	23	40
13	35	0	Placenta previa, Uterine atony	CS	36–2	8000	9	4	16	19	20
14	33	0	Placenta accreta spectrum	CS	41–2	4760	5	0	16	13	10
15	32	0	Amniotic fluid embolism (DIC-type), Uterine atony	CS	37–2	3800	18	8	30	21	40
16	41	0	Amniotic fluid embolism (DIC-type), Uterine atony	I-VD	38–4	7990	17	8	24	36	40
17	34	0	Placenta previa, Uterine atony	CS	32–2	3000	7	1	2	5	0
18	42	0	Placenta accreta spectrum	CS	40–6	1800	2	0	8	0	0
19	39	0	Amniotic fluid embolism (DIC-type), Uterine atony	CS	40–5	10,000	24	7	40	50	30
20	34	0	Placenta accreta spectrum	S-VD	40–0	4900	7	1	8	10	0
21	39	0	Amniotic fluid embolism (DIC-type), Uterine atony	CS	37–0	4600	12	6	18	20	10
22	39	1	Placenta accreta spectrum, Placenta previa	CS	36–1	1560	2	0	0	0	0
23	44	0	Amniotic fluid embolism (DIC-type), Uterine atony	CS	35–5	6150	9	6	18	5	10
24	32	0	Placenta previa, HELLP syndrome	CS	38–0	9600	23	8	38	59	90
25	45	0	Placenta previa, Uterine atony	CS	36–1	3500	8	3	4	8	0
26	31	0	Amniotic fluid embolism (DIC-type), Uterine atony	CS	35–2	6000	14	2	24	31	20
27	38	1	Pseudoaneurysm	CS	38–1	1300	0	0	0	0	0
28	38	0	Placenta accreta spectrum, Placenta previa	CS	36–5	1730	0	0	0	0	0
29	29	0	Uterine atony	S-VD	38–4	3090	8	1	12	11	
30	39	0	Retained products of conception	CS	35–1	2620	5	4	12	8	0
31	41	0	Placenta previa, Uterine atony	CS	36–1	2000	6	4	6	13	0
32	42	0	Amniotic fluid embolism (DIC-type), Placenta accreta spectrum, Placenta previa	CS	37–0	6500	11	6	30	35	30
33	38	1	Pseudoaneurysm	I-VD	37–2	1400	0	0	0	0	0
34	42	0	Retained products of conception	CS	38–5	2200	0	0	0	0	0
35	35	2	Placenta previa	CS	35–2	2160	3	0	0	0	0
36	43	3	Amniotic fluid embolism (DIC-type), Placental abruption	CS	39–0	6300	11	6	24	24	20
37	33	0	Amniotic fluid embolism (DIC-type)	CS	34–6	1700	16	5	10	19	10
38	34	0	Placenta previa, Uterine atony	CS	37–0	5433	10	2	14	23	30
39	41	0	Retained products of conception	CS	35–4	1800	3	2	4	4	0
40	38	0	Placenta accreta spectrum, Placenta previa	CS	36–1	1455	0	0	0	0	0
41	43	0	Placenta previa, Uterine atony	CS	35–4	1700	7	2	4	10	0

PPH, postpartum hemorrhage; GA, gestational age; CS, cesarean section; S-VD, spontaneous vaginal delivery; I-VD, instrumental vaginal delivery; DIC, disseminated intravascular coagulation; ISTH, International Society on Thrombosis and Hemostasis; RBC, red blood cells; FFP, fresh frozen plasma; PC, platelet concentrates.

**Table 2:** Findings during TAE.

Case	Embolized Vessels	Embolization Material	Complete Success	Efficacy of Hemostasis	Additional Procedure
1	UA, bilateral	gelatin	+	+	−
2	UA, bilateral Internal pudendal artery, right	gelatin	+	+	−
3	UA, bilateral	gelatin	+	+	−
4	UA, bilateral	gelatin	+	+	−
5	UA and internal iliac arteries, bilateral	gelatin	−	+	Re−embolization
	UA, internal iliac arteries, superior gluteal artery, and inferior gluteal artery, right	gelatin			
6	UA, bilateral	gelatin	+	+	−
7	UA, bilateral	gelatin	−	+	Re−embolization
	Inferior epigastric artery, right	gelatin, coil			
8	Internal pudendal artery and internal obturator artery, right	gelatin, coil	−	+	Hysterectomy
9	UA, bilateral	gelatin	+	+	−
10	UA and internal bladder arteries, bilateral	gelatin	+	+	−
11	UA, bilateral	gelatin	+	+	−
12	UA, bilateral	gelatin	+	+	−
13	UA, bilateral	gelatin	+	+	−
14	UA, bilateral	gelatin	+	+	−
15	UA, bilateral; internal iliac arteries, left	gelatin	+	+	−
16	UA and internal pudendal arteries, bilateral; internal obturator artery and inferior gluteal artery, right	NBCA	+	+	−
17	UA, bilateral	gelatin	+	+	−
18	UA, bilateral	gelatin	−	+	Hysterectomy
19	UA, bilateral Superior vesical artery and inferior vesical artery, left; superficial artery, right	NBCA	+	+	−
20	UA, bilateral	gelatin	+	+	−
21	UA, bilateral	NBCA	+	+	−
22	UA, bilateral	gelatin	+	+	−
23	UA, bilateral	gelatin	+	+	−
24	UA, bilateral	gelatin	−	+	Hysterectomy
25	UA, bilateral	gelatin	+	+	−
26	UA, bilateral	NBCA	+	+	−
27	UA, bilateral	gelatin	+	+	−
28	UA, bilateral Inferior epigastric artery, left; superior gluteal artery, right	gelatin, coil	+	+	−
29	UA and internal pudendal artery, bilateral	gelatin	+	+	−
30	UA, bilateral	gelatin	+	+	−
31	UA, bilateral	gelatin	+	+	−
32	UA, bilateral	NBCA	+	+	−
33	UA, bilateral	gelatin	+	+	−
34	UA, bilateral	gelatin	+	+	−
35	UA, bilateral	gelatin	+	+	−
36	UA, right	NBCA	+	+	−
37	UA and inferior epigastric artery, left	NBCA	+	+	−
38	UA, bilateral	gelatin	+	+	−
39	UA and ovarian artery, right	gelatin	+	+	−
40	UA, bilateral	gelatin	+	+	−
41	UA, bilateral	gelatin	+	+	−

UA, umbilical artery; NBCA, N-butyl-2-cyanoacrylate; +, Yes, −, No

**Table 3:** Comparison of the characteristics of patients with or without DIC.

	Obstetrical DIC Score			Obstetrical DIC Score			ISTH-DIC Score		
	≥8 (*n* = 19)	<8 (*n* = 22)	*p*-Value	≥13 (*n* = 9)	<13 (*n* = 32)	*p*-Value	≥5 (*n* = 11)	<5 (*n* = 30)	*p*-Value
Maternal age (years)	37.6	36.9	NS	35.2	37.9	NS	37.6	37.2	NS
BMI (kg/m^2^)	20.4	21.7	NS	21.2	21.1	NS	20.8	21.2	NS
Parity			NS			NS			NS
0	16 (80.5)	17 (77.3)		8 (88.9)	25 (78.1)		10 (90.9)	23 (76.7)	
≥1	3 (15.8)	5 (22.7)		1 (11.1)	7 (21.9)		1 (9.1)	7 (23.3)	
Mode of conception			0.021 *			NS			NS
Spontaneous	12 (63.2)	6 (27.3)		6 (66.7)	12 (37.5)		6 (54.4)	12 (40.0)	
Others	7 (36.8)	16 (72.7)		3 (33.3)	20 (62.5)		5 (45.5)	18 (60.0)	
Multiple gestation	3 (15.8)	1 (4.5)	NS	1 (11.1)	3 (9.4)	NS	1 (9.1)	3 (10.0)	NS
Number of prior CS			NS			NS			NS
0	17 (89.5)	18 (81.8)		8 (88.9)	27 (84.4)		11 (100.0)	24 (80.0)	
≥1	2 (10.5)	4 (18.2)		1 (11.1)	5 (15.6)		0 (0)	6 (20.0)	
Obstetric complications									
HDP	3 (15.8)	2 (9.1)	NS	3 (33.3)	2 (6.3)	0.028*	3 (27.3)	2 (6.7)	NS
GDM	2 (10.5)	2 (9.1)	NS	1 (11.1)	3 (9.4)	NS	1 (9.1)	3 (10.0)	NS

Data are shown as mean or number (%). * *p* < 0.05. NS, Not significant; BMI, body mass index; CS, cesarean section; HDP, hypertensive disorders of pregnancy; GDM, gestational diabetes mellitus; DIC, disseminated intravascular coagulation; ICTH, International Society on Thrombosis and Hemostasis.

**Table 4:** Comparison of the obstetric outcomes in patients with or without DIC.

	Obstetrical DIC Score			Obstetrical DIC Score			ISTH-DIC Score		
	≥8 (*n* = 19)	< 8 (*n* = 22)	*p*-Value	≥13 (*n* = 9)	<13 (*n* = 32)	*p*-Value	≥5 (*n* = 11)	<5 (*n* = 30)	*p*-value
GA at delivery (weeks)	36.8	36.9	NS	36.4	36.9	NS	37.4	36.6	NS
Mode of delivery			NS			NS			NS
S-VD	2 (10.5)	2 (9.1)		0 (0)	4 (12.5)		0 (0)	4 (13.3)	
I-VD	1 (5.3)	3 (13.6)		1 (11.1)	3 (9.4)		1 (9.1)	3 (10.0)	
CS	16 (84.2)	17 (77.3)		8 (88.9)	25 (78.1)		10 (90.9)	23 (76.7)	
Total blood loss (mL)	5729	2322	<0.001 *	6043	3220	0.006 *	5795	3119	0.041 *
<2000 mL	1 (5.3)	10 (45.5)		1 (11.1)	10 (31.3)		1 (9.1)	10 (33.3)	
≥2000 mL	7 (36.8)	12 (54.5)		2 (22.2)	17 (53.1)		4 (36.4)	15 (50.0)	
≥5000 mL	11 (57.9)	0 (0)		6 (66.7)	5 (15.6)		6 (54.5)	5 (16.7)	
Type of PPH			<0.001 *			NS			NS
Primary PPH	18 (94.7)	14 (63.6)		8 (88.9)	24 (75.0)		10 (90.9)	22 (73.3)	
Secondary PPH	1 (5.3)	8 (36.4)		1 (11.1)	8 (25.0)		1 (9.1)	8 (26.7)	
Transfusion (IU)									
RBC	20.8	4.8	<0.001 *	25.8	8	<0.001 *	24	7.4	<0.001 *
FFP	23.2	4.0	<0.001 *	30.0	7.6	<0.001 *	27.2	7.1	<0.001 *
PC	23.9	1.4	<0.001 *	34.4	4.8	<0.001 *	30.9	4.1	<0.001 *
FFP/RBC ratio	1.14	0.98	NS	1.17	1.03	NS	1.11	1.05	NS
Obstetrical DIC score	13.4	3.4	<0.001 *	17.2	5.2	<0.001 *	14.5	5.3	<0.001 *
ISTH DIC score	4.7	1.0	<0.001 *	5.4	1.8	<0.001 *	6.5	1.2	<0.001*

Data are shown as mean or number (%). * *p* < 0.05. NS, Not significant; GA, gestational age; S-VD, spontaneous vaginal delivery; I-VD, instrumental vaginal delivery; CS, cesarean section; PPH, postpartum hemorrhage; RBC, red blood cells; FFP, fresh frozen plasma; PC, platelet concentrates; DIC, disseminated intravascular coagulation; ISTH, International Society on Thrombosis and Hemostasis

**Table 5:** Comparison of the PPH etiology and embolic materials used for TAE in patients with or without DIC.

	Obstetrical DIC Score			Obstetrical DIC Score			ISTH-DIC Score		
	≥8 (*n* = 19)	<8 (*n* = 22)	*p*-Value	≥13 (*n* = 9)	<13 (*n* = 32)	*p*-Value	≥5 (*n* = 11)	<5 (*n* = 30)	*p*-Value
Etiology of PPH			<0.001 *			0.042 *			<0.001 *
AFE (DIC-type)	11 (57.9)	0		7 (77.8)	4 (12.5)		9 (81.8)	2 (6.7)	
Uterine atony	13 (68.4)	5 (22.7)		6 (66.7)	12 (37.5)		7 (63.6)	11 (36.7)	
RPOC	0	4 (18.2)		0	4 (12.5)		0	4 (13.3)	
Vaginal hematoma	0	1 (4.5)		0	1 (3.1)		0	1 (3.3)	
Placenta previa	7 (36.8)	8 (36.4)		2 (22.2)	13 (40.6)		2 (18.2)	13 (43.3)	
PAS	2 (10.5)	8 (36.4)		1 (11.1)	9 (28.1)		1 (9.1)	9 (30.0)	
Pseudoaneurysm	0	3 (13.6)		0	3 (9.4)		0	3 (10.0)	
Placental abruption	1 (5.3)	0		0	1 (3.1)		1 (9.1)	0	
Embolic materials			0.004 *			0.041 *			0.001 *
Gelatin	12 (63.2)	20 (90.9)		5 (55.6)	27 (84.3)		5 (45.5)	27 (90.0)	
NBCA	7 (36.8)	0		4 (44.4)	3 (9.4)		6 (54.5)	1 (3.3)	
Gelatin + Coil	0	2 (9.1)		0	2 (6.3)		0	2 (6.7)	

Data are shown as number (%). * *p* < 0.05. AFE, amniotic fluid embolism; RPOC, retained products of conception; PAS, placenta accreta spectrum; NBCA, N-butyl-2-cyanoacrylate; TAE, transarterial embolization; DIC, disseminated intravascular coagulation; ISTH, International Society on Thrombosis and Hemostasis

**Table 6:** Comparison of clinical outcomes of patients who underwent TAE for PPH complicated with or without DIC.

	Obstetrical DIC Score			Obstetrical DIC Score			ISTH-DIC Score		
	≥8 (*n* = 19)	<8 (*n* = 22)	OR (95% CI)	≥13 (*n* = 9)	<13 (*n* = 32)	OR (95% CI)	≥5 (*n* = 11)	<5 (*n* = 30)	OR (95% CI)
Complete success	16 (84.2)	20 (90.9)	0.53 (0.10–3.06)	6 (66.7)	30 (93.8)	0.13 (0.02–0.84)	9 (81.8)	27 (90.0)	0.50 (0.08–2.91)
Efficacy of hemostasis	19 (100.0)	22 (100.0)	–	9 (100.0)	32 (100.0)	–	11 (100.0)	30 (100.0)	–

Data are shown as number (%). OR, Odds ratio; CI, Confidence interval; TAE, transarterial embolization; DIC, disseminated intravascular coagulation; ISTH, International Society on Thrombosis and Hemostasis; –, Not applicable

## Data Availability

The data presented in this study are available on reasonable request from the corresponding author.
